# Excellent room temperature deformability in high strain rate regimes of magnesium alloy

**DOI:** 10.1038/s41598-017-19124-w

**Published:** 2018-01-12

**Authors:** Hidetoshi Somekawa, Alok Singh, Ryoji Sahara, Tadanobu Inoue

**Affiliations:** Research Center for Structural Materials, National Institute for Materials Science, 1-2-1 Sengen, Tsukuba, Ibaraki, 305-0047 Japan

## Abstract

Magnesium and its alloys have the lowest density among structural metallic materials; thus, this light-weight metal has great potential for reducing the weight, for example, of vehicles and trains. However, due to its crystal structure, deformability is poor; in particular, under compressive stress. In this study, we modified magnesium with bismuth as an alloying element, which has the characteristics of being likely to form precipitates instead of grain boundary segregation. The Mg-Bi binary alloy showed excellent deformability and high absorption of energy in high-strain rate regimes at room temperature via contribution of grain boundary sliding. These properties, which are closely comparable to those of conventional middle-strength aluminum alloys (Al-Mg and Al-Mg-Si series alloys), have never been observed before in magnesium alloys. The development of such properties opens the door for not only academic but also industrial research in magnesium.

## Introduction

When the weight of a vehicle is reduced by 100 kg, fuel consumption is estimated to improve by about 0.9 km/L^1^, making the use of light-weight material necessary from an ecological point of view. Magnesium has a low density of 1.74 g/cm^3^, which is much lower than that of conventional structural metallic materials, such as aluminum (2.70 g/cm^3^) and steel (7.87 g/cm^3^); thus, magnesium and its alloys are recognized as the next generation of light-weight metallic materials. Nevertheless, the application of magnesium and its alloys as structural parts is still limited, primarily because of poor deformability at room-temperature ranges. Shear fracture readily occurs at the beginning of plastic deformation, particularly under compressive stress. The deformation modes of magnesium and its alloys at room-temperature are dislocation slips on basal and prismatic planes as well as deformation twinning^2,3^. Although these deformation modes are controlled by the alloying elements and/or material processing, it has not still been obtained an effective method yet.

In addition to dislocation slips and deformation twinning, grain boundary sliding (GBS) is another common deformation mechanism, which leads to large elongation-to-failure and is observed at elevated temperature (more than approximately 0.5 *T*_m_, where *T*_m_ is the melting point) due to accommodation process including diffusion^4^. Here, it is really noticed that a reduction in temperature for occurrence of GBS is helpful to solve the problems of magnesium and its alloys. Interestingly, several studies have revealed that magnesium and some of its alloys exhibit room-temperature GBS, which can partially contribute to deformation^5–14^. Our recent studies have systematically investigated the effect of alloying elements on elongation-to-failure in tension at room-temperature using many different magnesium binary alloys^10^. Although most alloying elements play a role in preventing room-temperature GBS, two elements, viz., manganese and lithium, are likely to increase the contribution of GBS to deformation. In the quasi-static and low-strain rate regimes, a fine-grained Mg-Mn alloy exhibits reasonably good elongation-to-failure in tension and an accordion-like deformation in compression without any fractures^10,15^, which have never been observed in magnesium and its alloys.

However, two subjects need further evaluation for use as structural parts, such as in vehicles and trains; grain boundary segregation control and deformation rate/speed improvement. In the former case, most of the alloying elements, including manganese, segregate at grain boundaries via thermomechanical processes^10^; however, sites of grain boundary segregation by solute elements become favored as the origin of fractures and/or the crack-propagation with progressive plastic deformation. As for magnesium alloys, most solute elements are recognized to attribute not as grain boundary strengthening element but as grain boundary embrittlement element^16^. Grain boundary segregation is assumed to be an unsuitable method to further improve deformability. In the latter case, the strain rates and/or deformation speeds in all of the previous studies are in the quasi-static or low-strain rate regimes^10,15^. From the viewpoint of satisfaction with reliability and safety over a wide range of applications, it is necessary to improve such properties at not only quasi-static but also high-strain rate regimes. In this study, we achieved these points with an alloying element, which is unlikely to segregate at grain boundaries but plays a role in enhancing room-temperature GBS; especially the absorption energy is close to that of conventional middle-strength aluminum alloys.

## Results

### Deformability and absorption energy at high-strain rate regime

**Figure 1:**
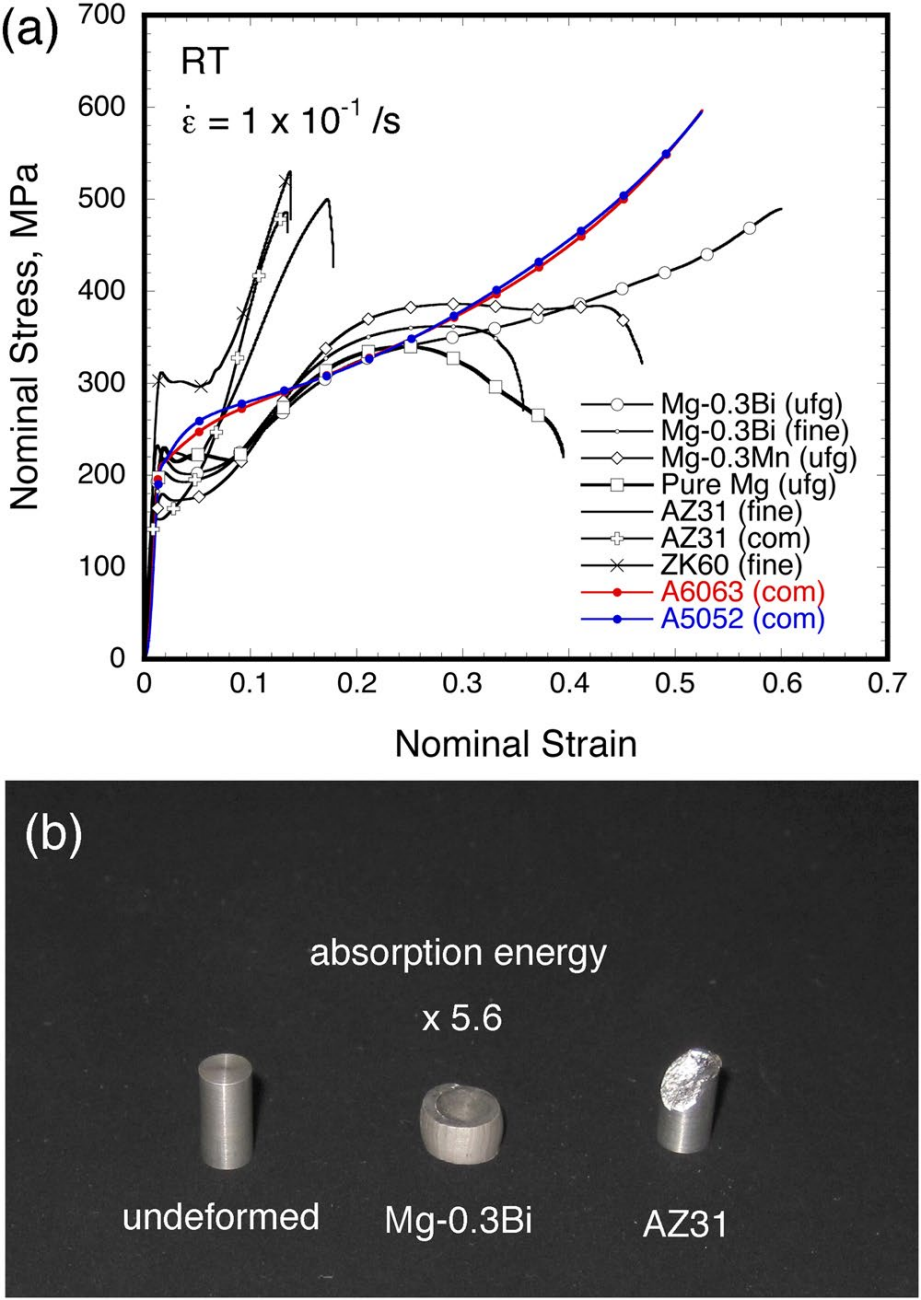
The results of mechanical properties for magnesium and its alloys at room temperature; (**a**) nominal stress vs. nominal strain curves at strain rate of 1 × 10^−1^/s obtained from compression tests, (**b**) appearance in specific magnesium alloy after the compression tests. Figure (**a**) includes the results of conventional middle-strength aluminum alloys (Al-Mg and Al-Mg-Si system alloys).

Nominal stress vs. nominal strain curves in the specific alloys and pure magnesium at a strain rate of 1 × 10^−1^/s obtained from compression tests are shown in Fig. 1(a). The stress vs. strain curves in all of materials are shown in Fig. S1(a). Figure 1(b) is the appearance of some of the magnesium alloys after compression tests. The stress vs. strain behavior clearly changes from alloy to alloy. A large strain hardening occurs after yielding, and then a rapid drop in load is observed in most of the alloys. The failure of conventional magnesium alloy (Mg-3mass%Al-1mass%Zn; AZ31), due to shear fracture as shown in Fig. 1(b), occurs at a nominal strain of less than 0.2. On the other hand, the Mg-Bi and Mg-Mn alloys are better deformability as compared to those of the commercial magnesium alloys. In particular, the ultra-fine-grained Mg-Bi alloy shows a constant stress, i.e., a plateau region, in compensation for such large strain hardening. In addition, it is difficult to make this Mg-Bi alloy fail or fracture, even up to a nominal strain of 0.6, i.e., good deformability at high-strain rate regime. The specimen after compression test is found to be barreled-shaped without any cracks, as observed in Fig. 1(b). The region enclosed by the stress vs. strain curve has a close relation to the absorption energy against fracture; to put the matter simply, the alloy with larger area under the curve exhibits a better ability to absorb energy before fracture. The absorption energy, *F*, is generally obtained from following equation;

1 $${\rm{F}}={\int }_{0}^{{{\rm{\varepsilon }}}_{{\rm{fail}}}}{\rm{\sigma }}({\rm{\varepsilon }}){\rm{d}}{\rm{\varepsilon }}$$

where ε_fail_ is the strain at failure. The absorption energy for AZ31 alloy is defined as 1 for ease of comparison. Figure 1(a) indicates that the ultra-fine-grained Mg-Bi alloy has at least 5.6 times higher absorption energy than that of AZ31 alloy. The comparison of absorption energy in the other alloys is summarized in Table S1. Stress vs. strain curves in the commercial extruded middle-strength aluminum alloys, i.e., A5053 and A6063 alloys, are included in this figure. The absorption energy of the ultra-fine-grained Mg-Bi alloy is close to that of these aluminum alloys, even in the high-strain rate regime. These results clearly reveal that the Mg-Bi alloy has excellent characteristics, i.e., large deformability and high absorption energy against fracture.

### Microstructures

**Figure 2:**
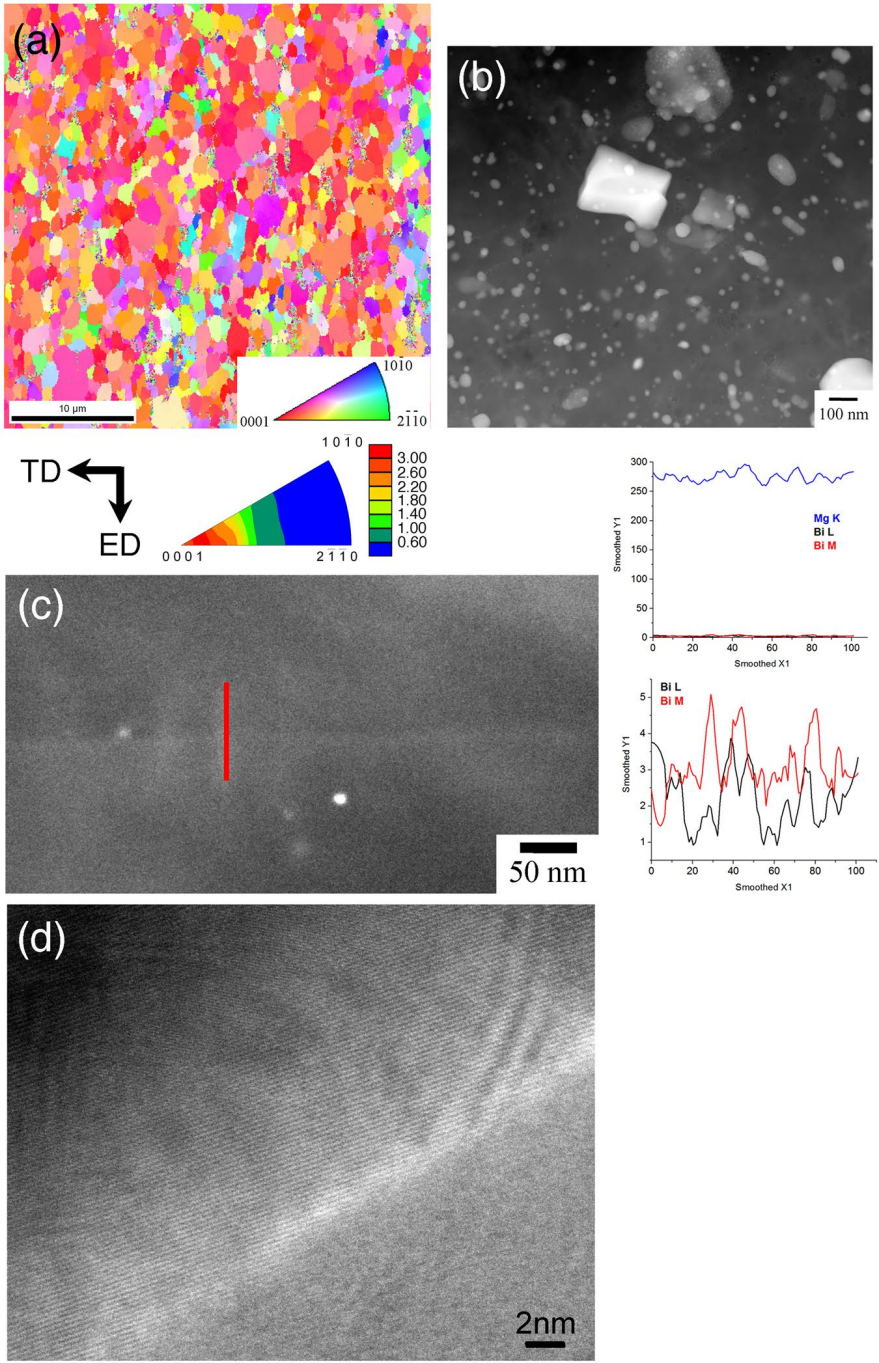
Initial microstructures of the ultra-fine-grained Mg-Bi alloy for (**a**) inverse pole figure image observed by EBSD method, (**b**) annular dark field (ADF) image taken by scanning transmission electron microscope (STEM), (**c**) STEM image containing a grain boundary and an EDX line-profile across it and (**d**) a lattice image of a grain showing its boundary by HREM (high resolution electron microscopy). TD and ED in Fig. (**a**) are transverse-direction and extrusion-direction, respectively.

The initial microstructures of the ultra-fine-grained Mg-Bi alloy extruded at 383 K are shown in Fig. 2 for (a) inverse pole figure image observed by electron back-scattering diffraction (EBSD), (b) annular dark field (ADF) image taken by transmission electron microscopy (TEM) and (c) a large magnification ADF image with energy-dispersive X-ray (EDX) line-profile across grain boundary. The microstructures of the other alloys are shown in Figs S2 and S3. Figure 2(a) shows that the ultra-fine-grained Mg-Bi alloy has recrystallized structures without any deformed microstructures, such as deformation twinning. The average grain size is obtained to be approximately 1 μm from the EBSD analysis software. The inverse pole figure from normal direction inset in Fig. 2(a) shows that this alloy also consists of a strong basal texture, i.e., the basal planes are parallel to the extrusion direction, because of production by hot extrusion. This basal texture is confirmed in the other binary alloys^10^ and is well-observed in most of the wrought processed magnesium alloys^17^. The images taken by TEM indicate that numerous precipitates of Mg_3_Bi_2_ are dispersed throughout the matrix (Fig. 2(b)). Figure 2(c) shows a grain boundary; the results of the EDX line-profile analysis indicate that bismuth element is unlikely to segregate at grain boundaries. In addition, Fig. 2(d) reveals the vicinity of grain boundaries observed by high-resolution electron microscopy (HREM). This alloy is found to have a clear/sharp grain boundary structure, i.e., the equilibrium grain boundary.

These microstructural features of the Mg-Bi alloy are different from those of other magnesium binary alloys. Grain boundary segregation is confirmed in most of wrought processed magnesium binary alloys, e.g., Mg-Ag, Mg-Ca, Mg-Mn, Mg-Sn, Mg-Zn and Mg-rare earth alloys^10,18–22^. In addition, when the chemical composition of the alloying element is 0.3 at.% in binary alloys (Mg-X alloy; where X = Al, Ag, Ca, Li, Mn, Pb, Sn, Y or Zn), precipitation is difficult to observe in the matrix even after thermomechanical processing^23^. Non existence of precipitates are due to the fact that the chemical composition of 0.3 at.% is much lower than the maximum solubility of the elements in magnesium^24^. In comparison with the other magnesium binary alloys, the Mg-Bi alloy has dispersed precipitates without any grain boundary segregation.

### Effect of alloying element on energetic feature

**Figure 3:**
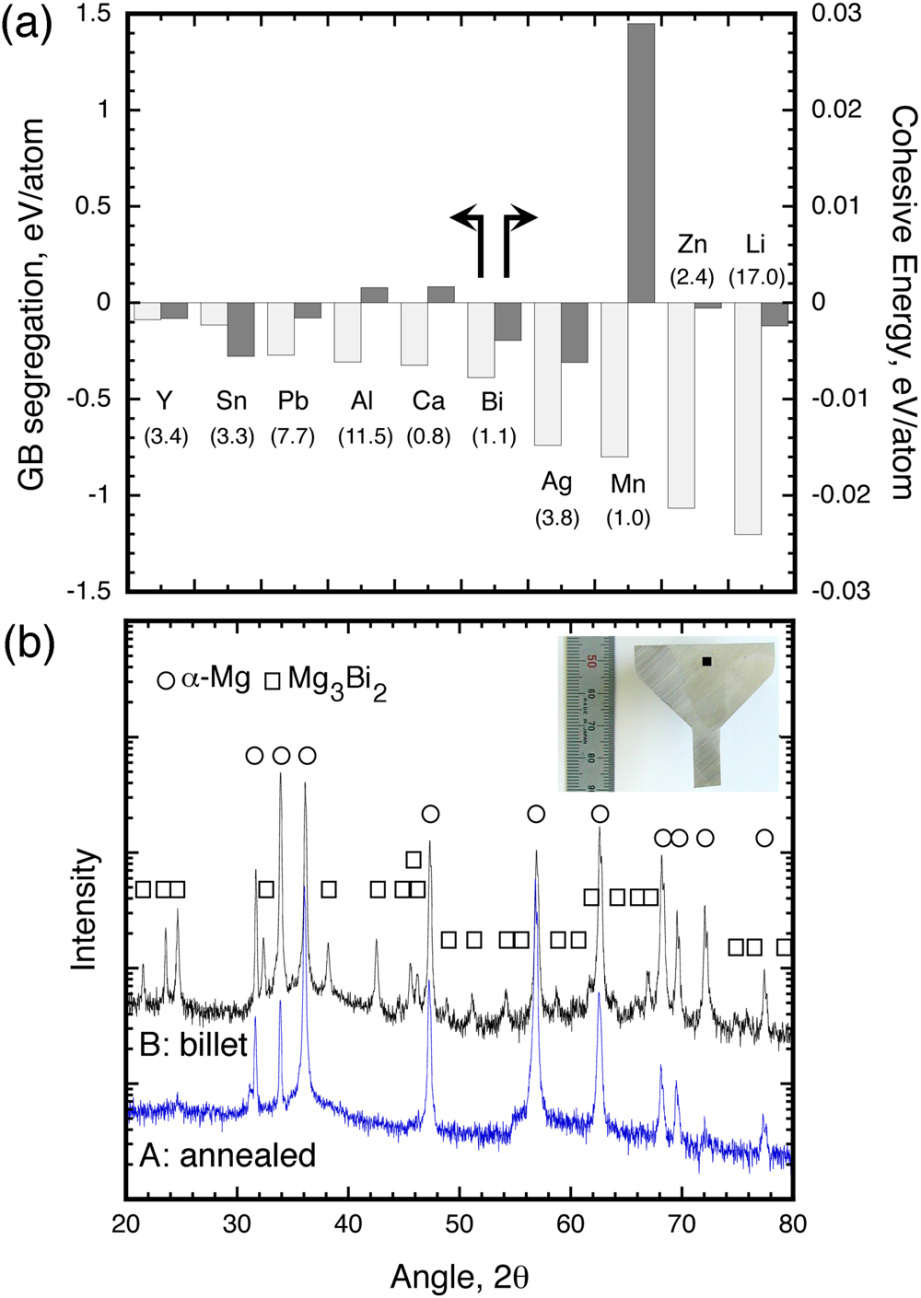
(**a**) grain boundary segregation energy and cohesive energy in each alloying models obtained from numerical analysis and (**b**) results of X-ray diffraction in the two kinds of Mg-Bi alloys, i.e., the annealed and before extruded Mg-Bi alloys. The values in parentheses in Fig. (**a**) are maximum solubility into magnesium^24.^

**Figure 4:**
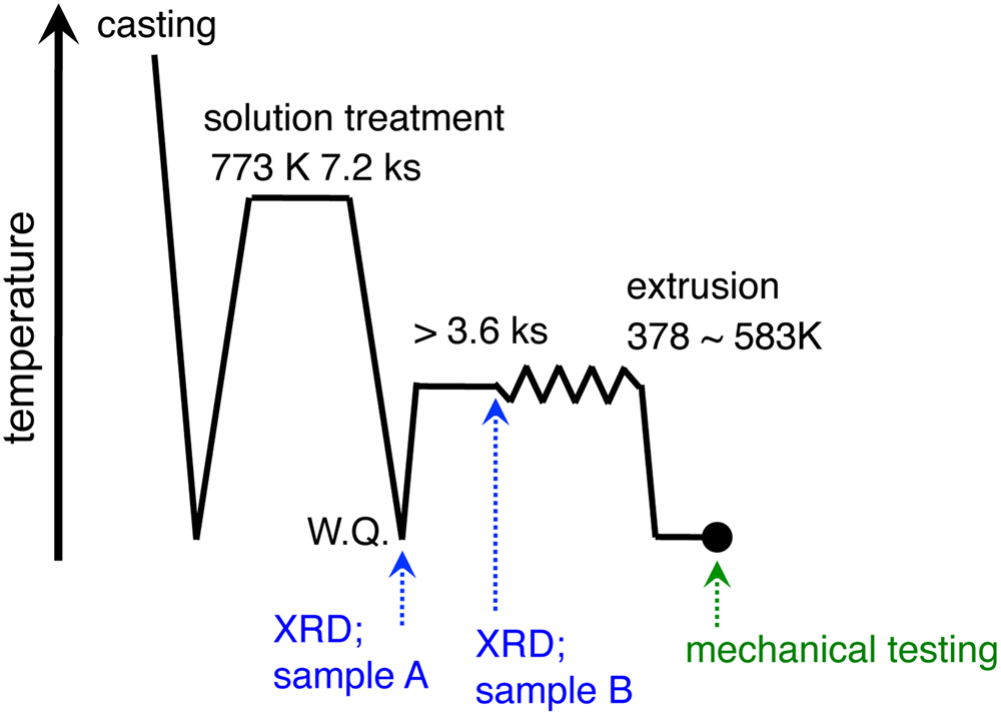
Schematic illustration of preparation procedure for magnesium binary alloys.

The reasons for these microstructural features in the Mg-Bi alloy are considered using numerical analysis. Grain boundary segregation energies of each alloying model are summarized in Fig. 3(a). The dependence of solute atom position on grain boundary segregation energies obtained from numerical analysis is provided in Fig. S4. In Fig. 3(a), all of the alloying atoms are found to have negative segregation energy values. This result indicates that solute atoms existing at grain boundaries are more stable as compared to those dispersed within the matrix. Except for the Mg-Bi model, the tendency for grain boundary segregation is consistent with the experimental results, observed by several microstructural methods, such as Z-contrast and three dimensional atom probe, as mentioned above. Figure 3(a) also includes the cohesive energies for crystalline alloy structures without any defects. Some of the models, i.e., the Mg-Mn, Mg-Al and Mg-Ca models, have positive energy values, but the other models have negative values. The positive and negative values indicate that solute atoms prefer to reside in the bulk structure and favor clustering, respectively. In the Mg-Bi model, it is interestingly noted that both grain boundary segregation energy and cohesive energy tend to have negative values. These results suggest that bismuth atoms are likely not only to segregate at grain boundaries but also to cluster, i.e., to form particles; however, Fig. 2(b),(c) show no grain boundary segregation. This is mainly related to the material processing; that is to say, the billet was kept in the furnace at the extrusion temperature before extrusion, as provided by preparation procedure in Fig. 4.

Figure 3(b) is X-ray diffraction (XRD) peaks of some of the Mg-Bi alloys, where the XRD measured samples are marked in Fig. 4. All of the peaks for the annealed Mg-Bi alloy before extruded billet preparation, correspond to the α-Mg phase; in contrast, the peaks for the alloy, which is un-extruded region marked by a black point inset right top-side in this figure, are confirmed to have both the α-Mg phase and the Mg_3_Bi_2_ phase. Thus, the precipitation particles form before the occurrence of grain boundary segregation. It is noted in Fig. 3(a) that silver and bismuth atoms have similar characteristics as alloying elements; nevertheless, the extruded Mg-Ag alloy show grain boundary segregation without any precipitation particles^10^. This results from the difference in solubility. While bismuth would like to segregate at grain boundaries, the solubility of bismuth into magnesium is a very low (close to zero) at the present extrusion temperatures of ~383–413 K^24^; as a result, most of the bismuth element, which exists in the matrix, form precipitates. An alternative solute element is assumed to have a characteristic, in which it i) is likely to segregate at grain boundary (=negative grain boundary segregation energy) and ii) has a low solubility at the thermomechanical processing temperature.

## Discussion

**Figure 5:**
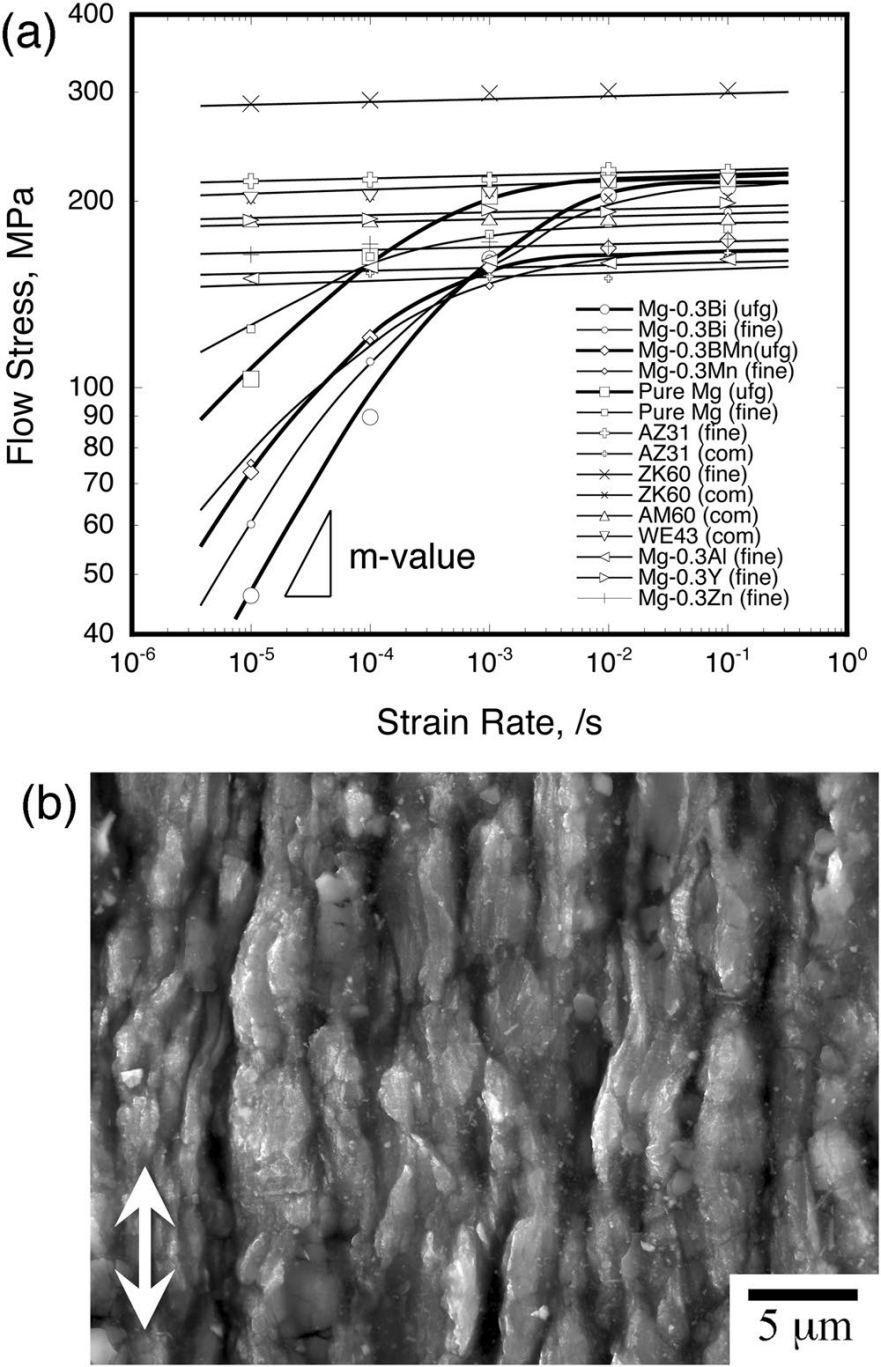
The analysis in compression tests at room temperature (**a**) flow stress as a function of strain rate and (**b**) typical deformed surface observation of the ultra-fine-grained Mg-Bi alloy at strain rate of 1 × 10^−2^/s. The white arrow in Fig. (**b**) indicates the compression direction.

The reason for attaining such excellent deformability and absorption energy in high-strain rate regimes of the Mg-Bi alloy is discussed, hereafter. The flow stress as a function of initial strain rate in compression tests is shown in Fig. 5(a). The flow stress is used in the nominal stress at the nominal strain of 0.025, as shown in Fig. S1. The flow stress is found to depend on the strain rate, and the slope of flow stress vs. strain rate corresponds to the strain rate sensitivity (*m*-value). This strain rate sensitivity well-reflects the plastic deformation mechanism in metallic materials^25^. When it is close to zero or is low, the dominant deformation mechanism is recognized as deformation twinning for *m* ≈ 0 and dislocation slip for *m* < 0.05 in HCP metals^25,26^. With an increase in this sensitivity (*m*-value), another deformation mechanism, i.e., GBS, is found to contribute to plastic deformation, irrespective of dislocation slips and deformation twinning. This figure shows that the strain rates in most magnesium alloys do not affect the flow stress; in contrast, the Mg-Bi alloys clearly have strain rate dependence. The strain rate sensitivities, as measured by the least-squares method, in high-strain rate regimes of this alloy and in each strain rate for all of the materials are listed in Table 1 and S2, respectively. Most of the alloys show low strain rate sensitivities of less than 0.05, which indicate that the rate-controlling mechanism is dislocation slips or deformation twinning. On the other hand, particularly, ultra-fine-grained Mg-Bi alloy has the *m*-value of more than 0.1, suggesting contribution of GBS, at the strain rate regimes of 1 × 10^−2^ and 10^−3^/s. A scanning electron microscopy (SEM) image of the deformed surface in the strain rate of 1 × 10^−2^/s of the ultra-fine-grained Mg-Bi alloy is shown in Fig. 5(b). The longitudinal direction is compression direction. Many traces of GBS are apparently observed in this image. These deformed features are confirmed in room-temperature tensile tested pure magnesium^8^ and Mg-Mn alloy^10^.

**Table 1:** List of extrusion temperature, average grain size and mechanical properties of the specific materials.

		*T*_ext_, K	*d*, μm	*F*	*m*-value*	*m*-value**
Mg-Bi	ultra-fine grained	383	1.2	5.6	0.04	0.11
Mg-Bi	fine grained	413	3.0	2.8	0.03	0.07
Mg-Mn	ultra-fine grained	478	1.4	4.0	0.02	0.03
Pure Mg	ultra-fine grained	378	1.4	2.9	<0.01	0.03
AZ31	fine grained	483	3.1	1.6	0.01	0.01
AZ31	commercial	—	19.1	1.0	<0.01	<0.01
ZK60	fine grained	473	3.0	1.3	<0.01	<0.01
A6063	commercial	—	—	6.1	—	—
A5052	commercial	—	—	6.0	—	—

where *T*_ext_ is the extrusion temperature, *d* is the average grain size and *F* indicates the absorption energy against fracture obtained from eq. (1) and defining 1.0 in the commercial AZ31 alloy. The symbols of * and ** in *m*-values are obtained in strain rate ranges, 10^−1^–10^−2^ s^−1^ and 10^−2^–10^−3^ s^−1^, respectively.

The contribution of GBS to deformation is also supported by the effect of alloying elements on flow stress. Pure magnesium and binary alloys studied here have similar average grain sizes of ~3 μm; thus, most of the binary alloys show higher flow stress than that of pure magnesium, because of solid solution strengthening. Regardless of the alloying element additions, Mg-Bi and Mg-Mn alloys show lower flow stress, indicating few or small contributions of dislocation slips to deformation. Hence, the excellent properties of the Mg-Bi alloy are due to the contribution of GBS, as is also the case for the Mg-Mn alloy.

In the Mg-Mn alloy, manganese element, which segregates at grain boundaries, plays an important role in enhancing GBS^10,15^. In the Mg-Bi alloy, although GBS also takes place despite the lack of grain boundary segregation, the Mg-Bi alloy is superior property. In ultra-fine-grained/fine-grained pure magnesium, GBS contributes to deformation without any grain boundary segregation^8^, as in the case of the Mg-Bi alloy. It is interestingly noted that, in the lower strain rate regimes, the flow stress of the Mg-Bi alloy is apparently lower than that of pure magnesium, although they have similar average grain sizes. It has been pointed out that GBS is influenced by the grain boundary structures^27–31^. The alloys having high fraction of non-equilibrium grain boundary structures tend to show lower strain rate for GBS occurrence. This is because long-range stress associated with the non-equilibrium grain boundaries having atomistic leveled facets and steps affects dislocation movement, which is the accommodation process of GBS. Such unique grain boundary structures have been confirmed in magnesium alloys produced by severe plastic deformation, e.g., equal-channel-angular extrusion, at relatively low temperature^31,32^. On the other hand, the microstructural observations, as shown in Fig. 2(c),(d), reveal that grain boundary characteristics of the Mg-Bi alloy are equilibrium grain boundaries. This mainly results from the presence of precipitation particles, which promotes dynamic recrystallization. It is necessary to further investigate the reasons behind such properties and microstructural features; however, despite no grain boundary segregation, the existence in clear/sharp grain boundaries, e.g., the formation of equilibrium grain boundary structures, is important for enhancing the deformability of magnesium and its alloys.

## Methods

### Experimental procedure

Fine-grained pure magnesium with a purity of 99.96 mass% and several fine-grained Mg-0.3at.X% (X = Al, Bi, Mn, Y or Zn) binary alloys were used in this study. The chemical compositions of the major alloying element and some conventional impurities in these binary alloys, which are measured by inductively coupled plasma mass spectrometry, are listed in Table S3. The reasons for selecting these alloying elements and chemical composition are that aluminum and zinc are common elements in magnesium alloys^33^, such as in Mg-Al-Zn and Mg-Zn-Zr system alloys, and rare-earth elements including yttrium are known to enhance non-basal dislocation slip activities^34,35^. When the grain size is about 3 μm, a minimum composition is estimated to be ~0.15 at.% in order to cover alloying elements in the vicinity of grain boundaries.

All of the binary alloys were produced by casting, and were then annealed at the temperature of 773 K for 2 hrs. These annealed alloys were extruded to control the average grain size to ~3 μm at various temperatures between 413 and 583 K in a rod-shape with a diameter of 8 mm (extrusion ratio of 25:1). Before extrusion, each billet was kept in the container for at least 1.8 ks at the extrusion temperature to reduce the differential temperature between the billet and the container. The material preparation procedure of these binary alloys is shown in Fig. 4. Comparisons were made with several commercial magnesium alloys (Mg-3Al-1Zn (AZ31), Mg-6Zn-0.5Zr (ZK60), Mg-6Al-0.5Mn (AM60) and Mg-4Y-3MM (WE43) alloys, in mass%) produced by extrusion. For some alloys, specifically Mg-Bi, Mg-Mn, AZ31 and ZK60 alloys, and pure magnesium, these materials with an ultra-fine-grained structure (~1 μm) and/or fine-grained structure (~3 μm) were also prepared by extrusion at a lower temperature. The extrusion temperatures and average grain sizes are summarized in Table S1.

The initial microstructures of pure magnesium and the alloys were observed by optical microscopy, EBSD, TEM (STEM) and HREM. The observations were made by optical microscopy and EBSD were the transverse-direction and the extrusion-direction for all of the materials. The samples for microstructural observations were prepared by mechanical polishing using SiC papers (#600, #800, #1200), diamond (6 and 1 μm) and alumina slurry, and then by chemical etching using an acidic-based solution. The samples for TEM and HREM observations were produced by thinning with an ion polishing system. The phases in two selected Mg-Bi alloys, as provided in Fig. 4, were identified by XRD using Cu-Kα radiation. One of these is the annealed alloy, i.e., before the extrusion billet preparation. The other is the non-severe plastic deformed region, as in the inset in Fig. 3(b).

The compression tests were carried out to investigate the deformability and the deformation mechanism at room-temperature. The initial strain rates were set to be 1 × 10^−1^, 10^−2^, 10^−3^, 10^−4^ and 10^−5^/s. The specimens were made by machining parallel to the extrusion direction, with a diameter of 4 mm and a height of 8 mm. The deformed sample after compression test at the strain rate of 1 × 10^−2^/s of the ultra-fine-grained Mg-Bi alloy was observed by SEM.

In order to compare room-temperature deformability in high-strain rate regime with the other competitive light-weight metallic materials, compression tests were carried out using commercial extruded aluminum 5052 and 6063 alloys (Al-2.41mass%Mg and Al-0.48mass%Mg-0.43mass%Si) with a diameter of 10 mm. The chemical compositions of the other elements are listed in Table S4. The method for specimen preparation and compression tests using aluminum alloys were the same as those as that described above for magnesium and its alloys. All of the compression tests were performed at least three times.

### Numerical procedure

First-principles calculations were performed to theoretically examine how alloying atoms are distributed at the boundary as well as within the bulk matrix, as shown in Fig. S5. Figure S5(a) provides schematic illustrations of the low-angle grain boundary model with 320 atoms, similar to our previous model^36^. Upper half regions are tiled by about 8.8°; so that, the periodicity for the <c> direction is satisfied. There are eighteen atoms at the central grain boundary, and they are labeled A through R, as shown in Fig. S5(b). One magnesium atom at the boundary is replaced by an alloying atom X (X* = *Ag, Al, Bi, Ca, Li, Mn, Pb, Sn, Y or Zn, which are able to sufficiently dissolve into magnesium in experiments^24^) to analyze the segregation energy. Since three-dimensional periodic boundary conditions were introduced, there are two interfaces in the model. The segregation energy is defined as;

2 $${E}_{Segregation}=[({E}_{bulk}^{(m+n){\rm{Mg}}}-{E}_{bulk}^{m\mathrm{Mg}+n{\rm{X}}})-({E}_{boundary}^{(m+n){\rm{Mg}}}-{E}_{boundary}^{m\mathrm{Mg}+n{\rm{X}}})]/n$$

where ${{\rm{E}}}_{{\rm{bulk}}}^{({\rm{m}}{\rm{+}}{\rm{n}})\mathrm{Mg}}$ is the total energy for the bulk (no boundary) with (*m* + *n*) Mg atoms, and *m* + *n* is 320. ${{\rm{E}}}_{{\rm{bulk}}}^{\mathrm{mMg}+\mathrm{nM}}$ is total energy for the bulk (no boundary) with *m* Mg and *n* X atoms for *n* = 1. The terms of ${{\rm{E}}}_{{\rm{boundary}}}^{({\rm{m}}{\rm{+}}{\rm{n}})\mathrm{Mg}}$ and ${{\rm{E}}}_{{\rm{boundary}}}^{\mathrm{mMg}+\mathrm{nM}}$ are the total energy for the boundary model with (*m* + *n*) Mg atoms and the total energy for the boundary model with *m* Mg and *n* X atoms, respectively. Using the model as shown in Fig. S5(c), the formation energy, which is described as the cohesive energy in the main text, in the bulk is also estimated by;

3 $${E}_{Formation}=(E({{\rm{Mg}}}_{m}{X}_{n})-m{E}^{{\rm{Mg}}}-n{E}^{X})/(m+n)$$

where *E*(Mg_*m*_X_*n*_) is the total energy of Mg_*m*_X_*n*_ alloy with *m* Mg and *n* X atoms, and *E*^Mg^ and *E*^*X*^ are the energies of Mg and X in their ground state phases, respectively, which are estimated with the Open Quantum Materials Database^37,38^ using *m* *=* 63 and *n* = 1.

In order to perform density functional theory (DFT) calculations, the projector augmented wave (PAW) method^39,40^, as implemented in the Vienna *Ab-initio* Simulation Package^39,40^ was used. The exchange-correlation energy was calculated within the generalized gradient approximation (GGA) by Perdew, Burke, and Ernzerhof^41^. The cut-off energy for the plane wave expansion was set at 400 eV. Electronic convergence was set as 10^−4^ eV for all cases. For the grain boundary model, Brillouin zone integrations were performed using a set of 18 × 12 × 4 k-points. Non-spin polarized calculation was considered in all the Mg-X models in this study. All of the atoms were fully relaxed except for those at the boundary. That is, for these atoms, atomic displacements for only the <*a*> and <*b*> axis were allowed to fix the plane of the grain boundary.

## Electronic supplementary material

supplementary information
