# Supplementary material for: Excellent room temperature deformability in high strain rate regimes of magnesium alloy
Source: Sci Rep. 2018 Jan 12;8:656. doi: 10.1038/s41598-017-19124-w (PMC5766617; doi:10.1038/s41598-017-19124-w)
Supplement: Supplementary file 1 — supplementary information [file 41598_2017_19124_MOESM1_ESM.doc]

# Excellent room temperature deformability in high strain rate regimes

# of magnesium alloy

Hidetoshi SOMEKAWA*, Alok SINGH, Ryoji SAHARA and Tadanobu INOUE

Research Center for Structural Materials, National Institute for Materials Science, 1-2-1 Sengen, Tsukuba, Ibaraki 305-0047 Japan

Supplementary

Supplementary Table S1: Summary of extrusion temperature, *T*_ext_, average grain size measured by linear intercept method, *d*, and ratio of absorption energy against commercial Mg-3mass%-1mass% (AZ31) alloy, *F*, obtained from eq. (1) in the main text.

|  |  | Text, K | d, μm | F |
| --- | --- | --- | --- | --- |
| Mg-Bi | ultra-fine grained | 383 | 1.2 | 5.6 |
| Mg-Bi | fine grained | 413 | 3.0 | 2.8 |
| Mg-Mn | ultra-fine grained | 478 | 1.4 | 4.0 |
| Mg-Mn | fine grained | 473 | 3.0 | 2.0 |
| Pure Mg | ultra-fine grained | 378 | 1.4 | 2.9 |
| Pure Mg | fine grained | 413 | 2.8 | 2.5 |
| AZ31 | fine grained | 483 | 3.1 | 1.6 |
| AZ31 | commercial | --- | 19.1 | 1.0 |
| ZK60 | fine grained | 473 | 3.0 | 1.3 |
| ZK60 | commercial | --- | 9.4 | 1.3 |
| AM60 | commercial | --- | 20.0 | 1.2 |
| WE43 | commercial | --- | 165 | 3.6 |
| Mg-Al | fine grained | 428 | 2.8 | 1.3 |
| Mg-Y | fine grained | 583 | 3.8 | 1.3 |
| Mg-Zn | fine grained | 443 | 2.7 | 1.4 |

Supplementary Table S2: The strain rate sensitivities (*m*-values), which are measured by the least square methods, in each strain rate of all the materials.

|  |  |  | m-value | |  |
| --- | --- | --- | --- | --- | --- |
|  |  | 10^-1^ - 10^-2^ /s | 10^-2^ - 10^-3^ /s | 10^-3^ - 10^-4^ /s | 10^-4^ - 10^-5^ /s |
| Mg-Bi | ultra-fine grained | 0.04 | 0.11 | 0.26 | 0.30 |
| Mg-Bi | fine grained | 0.03 | 0.07 | 0.17 | 0.26 |
| Mg-Mn | ultra-fine grained | 0.02 | 0.03 | 0.11 | 0.22 |
| Mg-Mn | fine grained | 0.01 | 0.04 | 0.09 | 0.20 |
| Pure Mg | ultra-fine grained | < 0.01 | 0.03 | 0.10 | 0.20 |
| Pure Mg | fine grained | < 0.01 | 0.02 | 0.04 | 0.11 |
| AZ31 | fine grained | 0.01 | 0.01 | < 0.01 | < 0.01 |
| AZ31 | commercial | < 0.01 | < 0.01 | < 0.01 | < 0.01 |
| ZK60 | fine grained | < 0.01 | < 0.01 | 0.01 | < 0.01 |
| ZK60 | commercial | < 0.01 | < 0.01 | < 0.01 | < 0.01 |
| AM60 | commercial | < 0.01 | < 0.01 | < 0.01 | < 0.01 |
| WE43 | commercial | < 0.01 | 0.02 | < 0.01 | < 0.01 |
| Mg-Al | fine grained | < 0.01 | < 0.01 | 0.01 | 0.02 |
| Mg-Y | fine grained | 0.01 | < 0.01 | 0.01 | < 0.01 |
| Mg-Zn | fine grained | 0.01 | < 0.01 | < 0.01 | 0.02 |

Supplementary Table S3: The chemical compositions measured by inductively coupled plasma mass spectrometry in magnesium binary alloys.

|  | X | Fe | Si | Mn | Ni |
| --- | --- | --- | --- | --- | --- |
| Mg-Al | 0.34 (0.30) | 0.002 | 0.002 | 0.003 | < 0.001 |
| Mg-Bi | 2.50 (0.30) | 0.004 | 0.002 | 0.003 | < 0.001 |
| Mg-Li | 0.09 (0.30) | 0.004 | 0.005 | 0.003 | < 0.001 |
| Mg-Mn | 0.65 (0.29) | 0.001 | 0.001 | 0.65 | < 0.001 |
| Mg-Y | 1.0 (0.27) | 0.003 | 0.003 | 0.003 | < 0.001 |
| Mg-Zn | 0.8 (0.29) | 0.004 | 0.002 | 0.003 | < 0.001 |

where the values are in weight % and those in parentheses indicate atomic %.

Supplementary Table S4: The chemical compositions in commercial extruded aluminum alloys.

|  | Mg | Si | Fe | Mn | Cu | Cr |
| --- | --- | --- | --- | --- | --- | --- |
| A6063 | 0.48 | 0.43 | 0.17 | 0.04 | < 0.01 | --- |
| A5052 | 2.41 | 0.04 | 0.18 | 0.02 | < 0.01 | 0.24 |

Supplementary Figure S1: The nominal stress vs. strain curves in compression tests of all of the materials; (a) initial strain rate of 1 × 10^-1^ /s, (b) 1 × 10^-2^ /s, (c) 1 × 10^-3^ /s, (d) 1 × 10^-4^ /s and (e) 1 × 10^-5^ /s. The curves of Mg-Al, Mg-Y and Mg-Zn are replotted by using previous our study [1]. Several black arrows indicate non-fracture.

[1] H. Somekawa, A. Singh and T. Inoue, Mater Trans. 58 (2017) 1089.

Supplementary Figure S2: The initial microstructures of extrusion-direction and transverse-direction for (a) commercial AZ31 alloy, (b) fine-grained AZ31 alloy, (c) commercial ZK60 alloy, (d) fine-grained ZK60 alloy, (e) commercial AM60 alloy and (f) commercial WE43 alloy. All of the images are taken by optical microscopy and are the same directions, as inset right top-side in Fig. (b).

Supplementary Figure S3: The initial microstructures for (a) ultra-fine-grained Mg-Mn alloy taken by EBSD, (b) fine-grained Mg-Bi alloy taken by EBSD and (c) TEM, respectively. TD and ED indicate transverse-direction and extrusion-direction, respectively.

Supplementary Figure S4: Grain boundary segregation energy obtained from numerical analysis vs. solute atom position in all of the alloying elements. The smallest value of each alloying model is used as grain boundary energy in main text, as shown in Fig. 3(a).

Supplementary Figure S5: The present models of numerical analysis for (a) grain boundary unit cell, (b) position of solute atom at grain boundary trough A to R and (c) bulk unit cell without any defects.
